# Treatment of Long Bone Fractures

**Published:** 1904-06

**Authors:** 


### TREATMENT OF LONG BONE FRACTURES.*

One of the most important chapters in surgery, and one to which too little attention is given in our medical schools to-day, is the subject of fractures. We can scarcely pick up a journal that does not contain some article on either gastric, gall tract or appendiceal surgery, but rarely one on fractures. We may judge from this that fractures are readily diagnosed and easily treated, and that the results are uniformly good.

*Read by Dr. M. E. Nuchols, Richmond, Va , Instructor in Surgery, University College of Medicine, before the Richmond Academy of Medicine and Surgery, March 22, 1[illegible]04.

How few of the graduates of our colleges know anything practical of the treatment of fractures, some of them never having seen a fracture, and yet how important it is that they should know. They may be called any day to treat a fracture, on the result of which their reputation may stand a living monument to their skill. On the other hand, they may never have to operate for perforating gastric ulcer or cholecystitis, and if they should their failure would be buried and soon forgotten.

The subject allotted to me is a very general one, and from this I judge that the gentlemen who selected the subject for me allowed me the privilege of choosing any part of it; at any rate I shall take the liberty and discuss, in a general way, the treatment of long bone fractures.

In order to be successful in the treatment we must necessarily be able to make a diagnosis of the bone fractured, the seat of fracture, the variety and extent of fracture and the associated lesion, if any exist.

A compound fracture or an intra-articular fracture requires somewhat different treatment from a simple fracture not involving a joint, and again, a fracture in an old person who has bronchitis, kidney trouble or some other constitutional disease, will need a different line of treatment from one in a healthy person. The treatment, therefore, resolves itself into treatment of the patient, treatment of the associated lesion and treatment of the fracture.

The thing to be considered when we first see a fracture is a dressing which will fix the parts and prevent further injury to the surrounding tissues and more extensive displacement. All fractures are attended by more or less laceration of periosteum, but rarely complete; a small strip remains connecting the two fragments called the periosteal bridge which is so necessary to the healing of the fracture, consequently every effort should be directed to preserve this by some fixed dressing.

It is not always advisable to do more than this in the beginning. The parts may be so much swollen, and the surrounding tissues so much contused, and muscular spasm so pronounced, as to render a diagnosis of the character and extent of the fracture uncertain, and successful reduction still less probable. Fortunately, this makes no material difference, as the work preparatory to union goes on just as well.

In compound fractures the primary treatment is addressed to the wound, only such dressing necessary to prevent injury to the surrounding tissues being used until healing is practically complete. The kind of treatment of the wound will depend on whether the fracture is produced by direct or indirect violence.

When produced by direct violence the soft parts are contused and lacerated to such an extent that the vitality of the tissues is very much impaired. These wounds are always infected, and sloughing usually occurs. The wound should be rendered as clean as possible, all fragments of bone being completely detached, removed and free drainage instituted. Healing takes place by granulation and is usually very slow.

If the fracture is produced by indirect violence, the broken bone being forced through the skin, or if by a gun-shot wound, the treatment is usually much simpler. Often the wound is not infected, and with ordinary care heals by first intention.

Compound fractures produced by modern bullets may be extensively comminuted, and some fragments may require removal.

Occasionally, on account of advanced age or general systemic disease, we may have to give the patient the first consideration, using all means at our command to save life and regarding the outcome of the fracture of little moment.

Whenever it is deemed advisable, the fracture should be reduced, still there are a few cases in which reduction is inadvisable, as in impacted fracture of the neck of the femur in the aged, and in others reduction is impossible, either on account of the interposition of muscle or contraction of muscles attached to the broken fragment, as the tuberosities of the humerus, trochanters of the femur and olecranon process. Reduction should be accomplished in these cases by operation after failure by other methods.

In reducing fractures it is necessary to have an accurate knowledge of the muscles in relation to the part, to know their attachment, action and power of contraction, and to overcome them by position and mechanical appliances.

In fractures of the shaft of the humerus and femur the proximal fragment is always rotated outward and lifted, hence the distal fragment must be placed in a corresponding position. The muscles attached to these bones are very powerful and overriding is frequent. To counteract this, extension and sometimes counter-extension is necessary. The amount of weight used in extension will depend on the size and degree of development of the muscles. The appliance used for extension in fractures of the femur will be wholly a matter of individual preference, Buck's extension being the most popular in this country. Hodgen's suspending splint is one of the best. Extension in children is usually made in the vertical position. The limb should always be put up in the position which best relaxes the muscles attached to the broken fragment, thus rendering subsequent displacement less liable to occur.

After reduction is accomplished, the simplest dressing which maintains apposition, gives perfect immobilization and allows free inspection of the parts, should be used. As a rule no dressing completely enveloping the fracture, such as plaster of paris bandages, should be used at first, because it interfers with circulation, is hard to remove, if such becomes necessary, and does not allow inspection of the seat of the fracture, which is important in order to detect recurrence of displacement. Moulded plaster of paris splints, however, are often very useful in the early treatment and makes one of the best splints. It is a good plan, I think, in many fractures, particularly Colle's, to remove the dressing from time to time to allow free inspection, in order to be assured that reduction is being maintained. I have used this plan in a number of instances and have never had occasion to regret it.

Fractures involving joints should not be immobilized too long, because a stiff joint is apt to result. The synovial membrane becomes inflamed, thickened and adhesions form. Callus is thrown out into the surrounding muscles, ligaments and capsule, and if allowed to become hard will result in ankylosis. After two or three weeks usually passive motion and massage should be used.

Fractures involving the shaft of long bones, such as the humerus and femur, require perfect immobilization for six or eight weeks, and in old people for a longer period. Unless this is adhered to strictly, fibrous union or non-union will occur. I do not hesitate to say that I am not much of a believer in the ambulatory treatment of fractures. I would recommend it for extremely old people where confinement in bed for a long period of time would mean death. Fracture of the neck of the femur, under these circumstances, after confinement to bed for about two weeks, could be treated by the Thomas hip splint. Just here I will say that I believe all fractures of the neck of the femur, it matters not how old the patient may be, should be treated and not regarded as hopeless. Many cases where union would least be suspected will recover with good results.

In separation of an epiphysis and diaphysis, reduction is absolutely essential to success. It is claimed by good authorities that if complete reduction is accomplished ossification is not apt to occur, and arrest of growth does not result, but if reduction is incomplete, ossification takes place with failure of growth.

Before concluding I will say a few words about the treatment of ununited fractures. Formerly it was thought necessary to wire the bones together in order to secure union; now we know that this is not only unnecessary, but usually objectionable, because the wire remains and may set up irritation. All that is necessary is to freshen the surfaces, bring them in apposition with as little disturbance to the periosteum as possible and retain them. Chromic catgut is used with best results, holding the fragments until sufficient callus is poured out to fix them, though no suture is necessary if the bones are placed in apposition and a plaster cast applied at once. Union is always slower, because there is no periosteal bridge, it taking place from the medullary canal and by granulation of the bone surfaces.—*Southern Medicine and Surgery.*

